# Auditory integration training techniques for brain stimulation to reduce attention deficit hyperactivity disorder symptoms and improve academic and behavioral skills among children with learning disabilities

**DOI:** 10.25122/jml-2025-0170

**Published:** 2026-02

**Authors:** Reem Alyoubi, Ahmed Issa, Hamza Al-Mwaulid, Anas Alyazidi

**Affiliations:** 1 Pediatric Neurology Section, Department of Pediatrics, Dr. Soliman Fakeeh Hospital, Jeddah, Saudi Arabia; 2 Department of Special Education, Arish University, North Sinai, Egypt; 3 Auditory Integration Training Program, Khadijah Attar Center, Dr. Soliman Fakeeh Hospital, Jeddah, Saudi Arabia; 4 Department of Instructional Technology and Design, College of Education, University of Jeddah, Jeddah, Saudi Arabia; 5 Pediatric Neurology Section, Department of Pediatrics, King Abdulaziz University Hospital, Jeddah, Saudi Arabia

**Keywords:** auditory integration training, ADHD, learning disabilities, sensory processing disorder, behavioral therapy, AIT, Auditory Integration Training, ADHD, Attention Deficit Hyperactivity Disorder, LD, Learning Disabilities, APD, Auditory Processing Disorder, DSM-5, Diagnostic and Statistical Manual of Mental Disorders, Fifth Edition, T0, Baseline, T1, Mid-intervention (Day 5), T2, Immediate Post-intervention (Day 10), T3, Follow-up (15 days after program completion), RCT, Randomized Controlled Trial

## Abstract

This study investigated the therapeutic effectiveness of the Berard Auditory Integration Training (AIT) protocol, a neurosensory intervention believed to address underlying auditory processing deficits that often contribute to the high comorbidity of learning disabilities (LD) and attention-deficit/hyperactivity disorder (ADHD), for treating children diagnosed with both conditions. A prospective, single-arm interventional study was conducted using a standardized 10-day AIT program with the Earducator device on ten pediatric participants with dual diagnoses. Measurements were taken at baseline, mid-intervention, post-intervention, and a 15-day follow-up using a validated behavioral observation checklist and an academic skills assessment. Quantitative analysis demonstrated marked improvements across all domains, with hyperactivity and attention-deficit scores significantly decreasing from severe to mild-moderate levels, and academic skills such as letter recognition and language showing consistent improvement. Furthermore, qualitative parental reports corroborated these findings, noting improvements in social interaction, reduced auditory sensitivity, and better adaptive behaviors. The findings provide preliminary evidence that AIT serves as an effective adjunctive therapy for reducing core ADHD symptoms and fostering academic and behavioral gains in children with LD, thus justifying the need for larger, randomized controlled trials.

## Introduction

The frequent comorbidity of learning disabilities (LD) and attention deficit hyperactivity disorder (ADHD) presents a significant clinical challenge, often leading to profound academic underachievement and behavioral dysregulation [1]. Research suggests that these conditions may share underlying neurobiological deficits, particularly in auditory processing. Auditory processing disorder (APD) is a common correlate, manifesting as difficulties in phonological awareness, auditory discrimination, and filtering extraneous sensory input, which directly impacts reading fluency and attentional control [2,3]. Auditory integration training (AIT), pioneered by Dr. Guy Berard, is a neurosensory intervention predicated on the theory that disordered auditory perception contributes to behavioral and learning pathologies [4]. The technique involves listening to electronically modulated music designed to train and normalize the auditory system, thereby improving the brain's ability to process sensory information. This, in turn, is hypothesized to enhance cognitive function, behavioral regulation, and learning capacity [4]. Prior research has indicated that musical and auditory interventions can positively influence language skills and auditory processing [5-7]. Furthermore, studies by Lauterbach [8] and Cancer *et al*. [9] have shown promising results for auditory training in populations with ADHD and dyslexia, respectively. However, the specific impact of the standardized Berard AIT protocol on the complex presentation of children with dual diagnoses of LD and ADHD remains insufficiently explored. This study aimed to fill this gap by evaluating the effect of AIT on a core set of clinical outcomes: ADHD symptoms, foundational academic skills, and adaptive behaviors.

## Material and Methods

### Study design and participant selection

A prospective, single-arm interventional study was conducted at the Khadijah Attar Center for Special Needs, affiliated with Dr. Soliman Fakeeh Hospital in Jeddah. A convenience sample of ten children (*n* = 10) with a confirmed dual diagnosis of LD and ADHD was recruited. Diagnosis was established by a consultant pediatric neurologist based on the Diagnostic and Statistical Manual of Mental Disorders, Fifth Edition (DSM-5) criteria and supported by comprehensive educational and psychological assessments.

### Intervention: Berard AIT protocol

The intervention consisted of the standardized Berard AIT protocol, delivered twice daily for 10 consecutive days (20 total sessions) using the Earducator device. Each 30-minute session was separated by a minimum 3-hour interval. The intervention involved participants listening to music processed in real time to modulate random frequencies and intensities through headphones. The sound intensity was progressively calibrated for each participant, starting at 55 dB and increasing to 75 dB, with individual adjustments made to filter out frequencies to which each participant showed hypersensitivity, as per the established protocol [4]. A sample training session configuration is detailed in the original document's Protocol (A).

### Outcome measures and assessment timeline

The primary tools for measuring outcomes were two researcher-administered checklists:

***Behavioral observation checklist:*** A 20-point scale evaluating 10 domains of behavior, including hyperactivity, attention deficit, physical aggression, sleep disturbances, and social interaction.

***Academic skills checklist:*** A 20-point scale evaluating 10 domains of pre-academic and academic function, including alphabet reading/writing, receptive/expressive language, vocabulary, and sound imitation.

Assessments were conducted at four critical time points to track progression: Baseline (T0), Mid-intervention (Day 5, T1), Immediate Post-intervention (Day 10, T2), and Follow-up (15 days after program completion, T3).

### Statistical analysis

Given the pilot nature of the study, data were analyzed using descriptive statistics to identify trends. Mean scores for each domain across the four assessment periods were calculated and compared to visualize the trajectory of change. Qualitative data from structured parental interviews were collected at T2 and T3 to triangulate and enrich the quantitative findings.

## Results

### Quantitative improvements in behavioral and academic domains

The data revealed a consistent improvement trend from T0 to T3 across nearly all measured variables. The most pronounced effects were observed in core ADHD symptoms and associated behavioral challenges.

**Figure 1:**
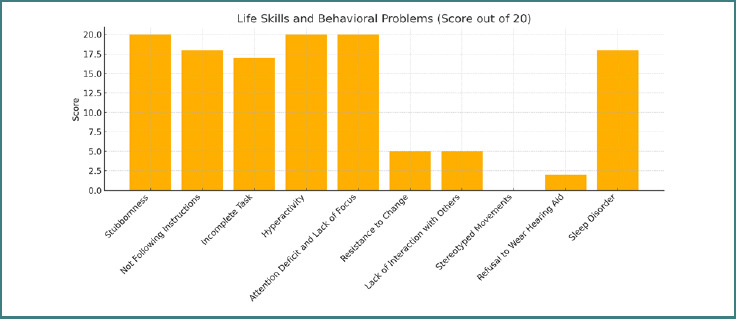
Illustration of life skills and behavioral issues

As demonstrated in Table 1, scores for hyperactivity and attention deficit showed a marked decline. For instance, the group's mean score for hyperactivity decreased from a baseline level indicative of severe impairment to a level reflecting mild to moderate impairment by the follow-up assessment. Similarly, behaviors such as physical aggression, non-compliance, and stereotyped movements were substantially reduced (Figure 1).

**Table 1:** Evaluation of behavioral problems and academic skills after 10 days of training

Life Skills and Behavioral Problems	Stubbornness	Not following instructions	Incomplete task	Hyperactivity	Attention deficit and lack of focus	Resistance to change	Lack of interaction with others	Stereotyped movements	Refusal to wear hearing aid	Sleep disorder
**Score** **(*n* = 20)**	20	18	17	20	20	5	5	–	2	18

**Figure 2:**
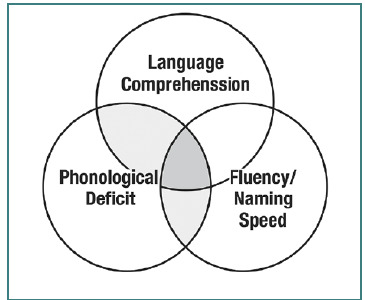
Primary types of developmental reading disabilities

Concurrently, significant gains were recorded in academic and language skills. Figure 2 provides a conceptual framework for these improvements, which were quantified in the academic checklists. Scores in receptive language, expressive language, and sound imitation showed the most robust increases. For example, the ability to follow multi-step instructions and articulate words clearly improved notably across the cohort, as reflected in the rising scores from T0 to T3.

### Longitudinal outcomes and follow-up

The positive trajectory observed at T2 was largely maintained or even improved upon the 15-day follow-up (T3), suggesting that the benefits of AIT may extend beyond the immediate intervention period. Table 2 consolidates this data, showing sustained or enhanced performance in areas such as letter writing, task attention, and social interaction.

**Table 2:** Evaluation of academic and behavioral issues 15 days after completing the program

Academic and behavioral skills	Letter reading	Letter writing	Coloring outlined shapes and copying	Receptive language and following instructions	Reading difficulties (words & sentences)	Expressive language	Aggression and self-harm	Attention deficit and hyperactivity	Sleep disorder	Interaction with others
Case 1	16	15	16	14	8	17	10	6	13	16
Case 2	17	18	15	18	7	17	9	9	14	16
Case 3	17	18	14	18	9	19	14	11	11	17
Case 4	19	18	17	19	9	19	15	12	9	17
Case 5	19	19	18	18	6	18	8	13	8	17
Case 6	19	19	17	19	8	19	9	7	9	17
Case 7	19	19	15	18	12	18	14	8	12	16
Case 8	17	17	11	15	11	17	16	9	5	17
Case 9	11	14	9	15	8	19	12	7	9	17
Case 10	16	16	11	15	11	18	15	13	12	18

### Qualitative corroboration from parental reports

**Figure 3:**
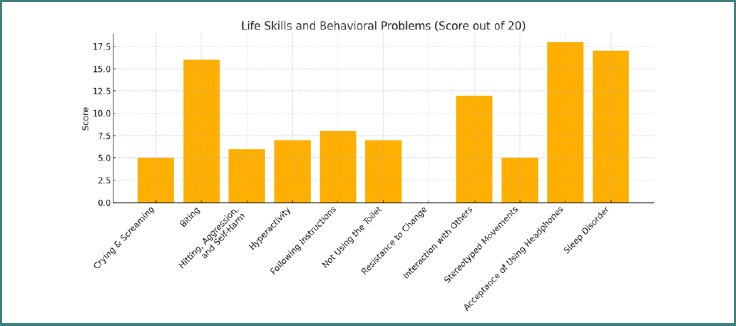
Evaluation of behavioral issues and academic skills by parental observation

Structured interviews with parents provided compelling qualitative evidence that supported the quantitative data (Figure 3). Commonly reported observations included:

- A notable increase in the child's attention span and ability to sit for extended periods during tasks.
- A clear reduction in auditory hypersensitivity; for example, several parents reported that their children no longer cover their ears in response to everyday household noises, such as blenders.
- Enhanced social communication, including initiation of greetings, better eye contact, and appropriate responses to questions.
- Improvements in adaptive life skills, such as properly wearing shoes and clothing, and accepting a wider variety of foods.

These reports align closely with the improvements quantified in the behavioral checklists, particularly in the domains of social interaction, resistance to change, and sleep disorders.

## Discussion

This pilot study demonstrates that the Berard AIT protocol was associated with multi-faceted improvements in children with LD and ADHD. The reduction in core ADHD symptoms—hyperactivity, impulsivity, and inattention—suggests that AIT may exert a modulating effect on neural systems governing arousal and self-regulation. This is consistent with the findings of Tremblay & Kraus [10], who proposed that AIT modifies neural representations of sound, thereby improving the efficiency of information processing in working memory, a domain frequently impaired in ADHD.

The concurrent gains in academic skills, particularly in language and pre-literacy areas, can be interpreted as a downstream effect of improved auditory processing. By "retraining" the auditory system to process phonemes more accurately and filter out irrelevant sensory input, AIT may reduce the cognitive load associated with listening and decoding language. This freed-up cognitive capacity can then be allocated to higher-order learning tasks, a phenomenon supported by Tierney & Kraus [11] and by our study's academic assessments. The positive changes in social behavior and adaptive skills reported by parents further underscore the interconnectedness of sensory processing, behavior, and learning. As hypothesized by Berard and observed in our cohort, when auditory distortions and hypersensitivity are reduced ("hearing equals behavior"), children become less distressed, more available for social engagement, and more receptive to learning from their environment [4]. The reduction in sleep disturbances in over six cases is a particularly noteworthy finding, suggesting a potential regulatory impact on the autonomic nervous system.

### Clinical implications

For clinicians seeking multi-modal treatment approaches for complex neurodevelopmental disorders, AIT presents a promising, non-pharmacological adjunctive therapy. Its structured, short-duration protocol is practical for implementation in clinical settings. The findings suggest that AIT could be particularly beneficial for children with LD/ADHD who present with signs of auditory sensitivity or processing delays.

Future research must employ rigorous randomized controlled trials (RCTs) with larger, more diverse cohorts. The incorporation of objective outcome measures, such as standardized ADHD rating scales (e.g., Conners' Scale), electrophysiological testing (e.g., Auditory Brainstem Response), and functional neuroimaging, would provide more robust and mechanistic evidence of AIT's efficacy.

### Limitations

The conclusions of this study are tempered by its limitations, including the small sample size, the absence of a control group, and the use of non-blinded assessments. These factors limit the ability to make causal inferences or entirely rule out placebo or maturation effects.

## Conclusion

In this cohort of children with learning disabilities and comorbid ADHD, the Berard Auditory Integration Training protocol was associated with statistically and clinically significant improvements in behavioral regulation, academic proficiency, and social adaptive functioning. The intervention was well-tolerated, and benefits appeared to persist beyond the active treatment period. These compelling pilot results provide a strong rationale for conducting larger, controlled studies to definitively establish AIT as an evidence-based intervention within the integrated clinical management of complex neurodevelopmental disorders.

## Data Availability

Further data is available from the corresponding author upon reasonable request.
